# Association Between Composite Dietary Antioxidant Index and Asthma‐Chronic Obstructive Pulmonary Disease Overlap Syndrome: Evidence From a Large Cohort in the UK Biobank

**DOI:** 10.1002/fsn3.72153

**Published:** 2026-07-26

**Authors:** Chunli Zeng, Hequn Lyu, Lihong Lou, Xixue Lu, Ming Lei

**Affiliations:** ^1^ Department of Critical Care Medicine Seventh People's Hospital of Shanghai University of Traditional Chinese Medicine Shanghai China; ^2^ Department of Lung Disease Yancheng TCM Hospital Affiliated to Nanjing University of Chinese Medicine Yancheng China; ^3^ Department of Acupuncture and Rehabilitation Affiliated Hospital of Nanjing University of Chinese Medicine Nanjing China; ^4^ Department of Acupuncture Yancheng TCM Hospital Affiliated to Nanjing University of Chinese Medicine Yancheng China; ^5^ Department of Acupuncture Affiliated Hospital of Traditional Chinese Medicine, Shandong First Medical University Jinan China

**Keywords:** ACOS, CDAI, cohort study, nonlinear, UK Biobank

## Abstract

Research on the association of the composite dietary antioxidant index (CDAI) with asthma‐chronic obstructive pulmonary disease overlap syndrome (ACOS) has not yet been reported. This study aimed to explore the association between CDAI and ACOS through a large‐scale cohort study from the UK Biobank. The baseline population was divided into three groups based on the tertiles of CDAI. Three Cox proportional hazards models were constructed to explore the relationship between CDAI and ACOS. Restricted cubic splines (RCS) were constructed to explore the presence of nonlinear relationships, with inflection points calculated by a recursive algorithm. Kaplan–Meier methods were employed to investigate the cumulative risk of outcomes over time. Finally, subgroup analysis and sensitivity analysis were performed. After adjusting for all confounding factors, no significant association was found between CDAI and ACOS [1.01 (0.94, 1.09)]. Further RCS analysis revealed a nonlinear association, which may explain why the overall results were not statistically significant. The CDAI and ACOS exhibited an inverse association before and after the inflection point. The association was negative before and positive after the inflection point. Further stratified analysis revealed that the nonlinear association was only present in females, participants aged ≥ 60, and those with BMI < 30. No significant interactions were found in other subgroups. This large‐scale cohort study revealed a nonlinear association between CDAI and ACOS, and further identified differences based on gender, age, and BMI.

## Graphical abstract

In this prospective UK Biobank cohort study of 65,882 participants, we investigated the association between the Composite Dietary Antioxidant Index (CDAI) and incident asthma–COPD overlap syndrome (ACOS). During a median follow‐up of 12.45 years, CDAI demonstrated a nonlinear association with ACOS risk. Threshold analysis identified an inflection point at CDAI = 0.2, suggesting a potential threshold‐dependent relationship between dietary antioxidant status and ACOS development.

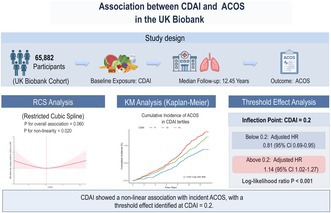

## 1 Introduction

Asthma and chronic obstructive pulmonary disease (COPD) rank among the most common chronic respiratory diseases worldwide, posing a significant burden on public health (Porsbjerg et al. 2023; Christenson et al. 2022). As the global population ages, the prevalence of asthma and COPD is on the rise (Wang et al. 2025; Collaborators GBDCRD 2020). When an individual patient exhibits characteristics of both asthma and COPD, asthma‐chronic obstructive pulmonary disease overlap syndrome (ACOS) may develop. Patients with ACOS typically face poorer long‐term conditions, characterized by more frequent and severe acute exacerbations of respiratory symptoms and accelerated lung function decline (Thomson 2017; Park, Kim, et al. 2018), leading to a sharp increase in mortality and healthcare costs (Park, Lee, et al. 2018; Fangal et al. 2024).

Oxidative stress plays a central role in the pathogenesis of ACOS. Oxidative stress refers to an imbalance between the production of reactive oxygen species (ROS) and the body's antioxidant defense system (Sies et al. 2017; Korovila et al. 2017). In patients with ACOS, the activation of inflammatory cells, exposure to environmental pollutants, and recurrent airway inflammation lead to excessive ROS production, exceeding the clearance capacity of the body's endogenous antioxidant system (Michaeloudes et al. 2022; Kirkham and Barnes 2013). This oxidative‐antioxidant imbalance contributes to exacerbated airway inflammation and remodeling, as well as disease progression (Hudler et al. 2022; Papaiwannou et al. 2014). Therefore, enhancing the body's antioxidant capacity may play a significant role in the reduced risk of these chronic respiratory diseases, warranting further investigation.

Dietary antioxidants, as an important component of the exogenous antioxidant defense system, have garnered increasing attention in recent years. Numerous epidemiological studies have shown that dietary micronutrients rich in antioxidants are associated with reduced risks of respiratory diseases (Bedard et al. 2018; Sdona et al. 2022; Islam et al. 2022; Allen et al. 2009). However, previous studies have primarily focused on the effects of individual antioxidant nutrients, and the synergistic interactions between different antioxidant compounds and their collective impact on health outcomes may be more clinically relevant than the effects of individual nutrients. The composite dietary antioxidant index (CDAI) is a comprehensive measurement method that captures the combined antioxidant capacity of multiple dietary micronutrients (Zhao et al. 2022). This index has been widely applied in various fields, including cardiovascular disease, cancer, and metabolic disorders (Liu, Lai, et al. 2023; Yu et al. 2022; He et al. 2025).

Current research on the association between CDAI and respiratory diseases remains relatively limited. A cross‐sectional study based on the National Health and Nutrition Examination Survey found that higher CDAI scores were associated with improved lung function (Zheng et al. 2024). Another study indicated that a high CDAI dietary pattern may reduce the risk of COPD by alleviating systemic inflammatory responses (Liu, Li, et al. 2023). However, these studies have limitations such as small sample sizes and a predominance of cross‐sectional designs, and there is a lack of studies that conduct in‐depth analyses of ACOS. Given the important role of oxidative stress in the pathogenesis of ACOS, as well as the potential protective effects of dietary antioxidants, a systematic assessment of the association between CDAI and ACOS holds significant scientific and clinical value. This study aims to utilize large‐scale prospective cohort data to comprehensively explore the association between CDAI and the risk of ACOS, with the goal of providing scientific evidence for the development of dietary antioxidant‐based preventive measures for respiratory diseases and offering new insights for individualized nutritional interventions.

## 2 Methods and Materials

### 2.1 Study Population

**FIGURE 1:**
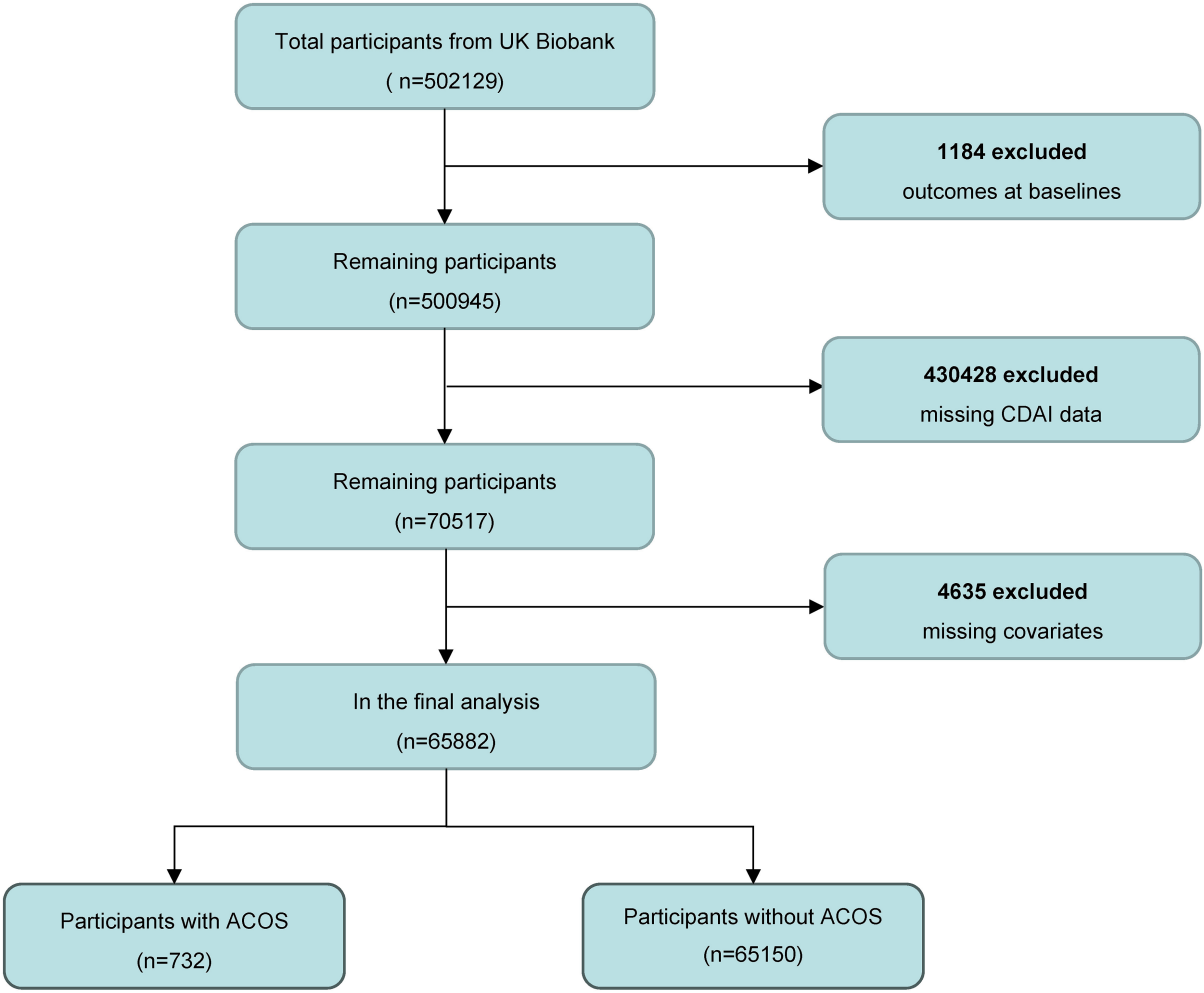
Flow chart of sample selection from UK Biobank.

The data for this study were obtained from the UK Biobank. The UK Biobank is currently one of the largest and most comprehensive long‐term prospective cohorts in the world, aimed at investigating the risk factors and mechanisms underlying major chronic diseases in middle‐aged and older adults. Between 2006 and 2010, the study recruited over 502,000 UK residents. Baseline data were collected through standardized questionnaires and clinical assessments, including sociodemographic characteristics, lifestyle factors, medical history, and physical measurements, along with blood, urine, and saliva samples for biochemical and genetic analysis. Some participants provided genotyping data and multimodal imaging data. The study linked with the UK National Health Service to access hospital records, primary care data, cancer registries, and death registries, enabling long‐term follow‐up and continuous updating of outcome information. The UK Biobank has been approved by the North West Multi‐Centre Research Ethics Committee, and all participants signed written informed consent forms. After excluding baseline outcomes, missing exposures, and missing covariates, the study included 65,882 participants (Figure 1).

### 2.2 Assessment of CDAI

Dietary data were collected using the Oxford WebQ system. This web‐based 24‐h dietary assessment tool was used for the first time at baseline. The assessment calculated energy and nutrient intake by multiplying the frequency of consumption of each food and beverage by standard serving sizes and nutritional content. The CDAI was calculated using methods described in previously published studies and included six dietary nutrients with antioxidant properties: vitamin A, vitamin C, vitamin E, selenium, zinc, and carotene (Zhang et al. 2024). The intake of each nutrient was first standardized based on the mean and standard deviation of the study population—that is, by subtracting the population mean from the individual intake and then dividing by the population standard deviation. The standardized values for each antioxidant nutrient are then summed to obtain each participant's total CDAI score. A higher CDAI value indicated greater overall antioxidant capacity from dietary sources. The specific calculation formula is as follows:

$$\text{CDAI}=\sum_{i=1}^{6}\frac{X_{i}-\mu_{i}}{\sigma_{i}}$$

where $X_{i}$ denotes the individual intake of each antioxidant component, and $\mu_{i}$ and $\sigma_{i}$ represent the corresponding mean and standard deviation, respectively.

### 2.3 Outcomes

The outcomes of this study were derived from an analysis of hospital admission records, which included admission and diagnosis data obtained from the English Hospital Incident Statistics System, the Scottish Morbidity Record Database, and the Welsh Patient Incident Database. Diagnoses were recorded according to the International Classification of Diseases, 10th Revision (ICD‐10) coding standards. ACOS diagnosis combined asthma (J45) and COPD (J41–J44) (Li et al. 2024). Follow‐up periods were calculated from the date of the baseline dietary assessment until the first outcome diagnosis, death, loss to follow‐up, or October 30, 2022, whichever occurred first.

### 2.4 Covariates

To control for the influence of confounding factors on study results, we included multiple levels of covariates. Demographic factors included age, gender, ethnicity, education, and the Townsend Deprivation Index. Lifestyle factors included smoking, drinking, physical activity, sleep scores, and dietary scores. The dietary scores were calculated by summing the scores of individual dietary components that met the recommended intake targets. One point was assigned for each component fulfilling the corresponding criterion, with higher scores indicating a healthier dietary pattern. BMI was included as a body measurement indicator. C‐reactive protein, white blood cell count (WBC), and neutrophils were included as inflammatory markers for adjustment. Chronic disease status included hypertension, hyperlipidemia, and ischemic heart disease. Forced expiratory volume in 1 s (FEV1) and forced vital capacity (FVC) were included as respiratory function indicators.

### 2.5 Statistical Analysis

The baseline population was divided into three groups based on the CDAI tertiles. Continuous variables were expressed as the median ± standard deviation or medians and interquartile ranges (IQR). Categorical variables were expressed as numbers and percentages. To explore the association of CDAI with ACOS, we constructed multivariate Cox proportional hazards models, with results presented as hazard ratios (HR) and 95% confidence intervals (CI). The results of proportional hazards assumptions were shown in Figure S1. To validate the presence of nonlinear relationships, restricted cubic splines (RCS) were constructed, and inflection points were calculated using a recursive algorithm. Two‐segment regression equations were used to assess the effect relationships before and after the inflection points. Kaplan–Meier methods were employed to investigate the cumulative risk of outcomes over time, and the log‐rank test was used to compare different groups. We conducted a stratified analysis by gender to explore gender‐related differences and used interaction tests to explore whether there were differences in other subgroups to further explore the stability of the study results. Finally, the sensitivity analysis was conducted to exclude participants who had experienced ACOS within 1–2 years of follow‐up and those with extreme CDAI, in order to verify the robustness of the study results. All statistical analyses were performed using R software, and a two‐sided *p* value of < 0.05 was considered statistically significant.

## 3 Results

### 3.1 Characteristics of the Study Population

Table 1 presents the characteristics of the enrolled population. In this cohort study, the median follow‐up duration was 12.45 (1.64) years for ACOS. The average age of the population was 56.19 (8.16) years, with 55.04% being female and 94.04% being white. Compared with other groups, the CDAI group at T3 was more likely to include former smokers who had quit, individuals who engaged in physical activity, and those with a college education. They typically had higher dietary scores, FEV1, and FVC. The CDAI group at T2 had the lowest risk of hypertension, hyperlipidemia, and ACOS.

**TABLE 1:** The characteristics of participants.

	Total	Tertile 1	Tertile 2	Tertile 3	*p* ^b^
	*N* = 65,882^a^	*N* = 21,961; < −1.82	*N* = 21,960; −1.82 to −1.01	*N* = 21,961; > 1.01	
Age					
Mean (SD)	56.19 (8.16)	55.76 (8.16)	56.42 (8.07)	56.39 (8.21)	< 0.001
Gender					
Female	36,259.00 (55.04%)	12,056.00 (54.90%)	11,972.00 (54.52%)	12,231.00 (55.69%)	0.041
Male	29,623.00 (44.96%)	9905.00 (45.10%)	9988.00 (45.48%)	9730.00 (44.31%)	
Ethnicity					
Non‐White	3929.00 (5.96%)	1669.00 (7.60%)	1045.00 (4.76%)	1215.00 (5.53%)	< 0.001
White	61,953.00 (94.04%)	20,292.00 (92.40%)	20,915.00 (95.24%)	20,746.00 (94.47%)	
Drinking status					
Never	2397.00 (3.64%)	891.00 (4.06%)	736.00 (3.35%)	770.00 (3.51%)	< 0.001
Previous	2230.00 (3.38%)	810.00 (3.69%)	637.00 (2.90%)	783.00 (3.57%)	
Current	61,255.00 (92.98%)	20,260.00 (92.25%)	20,587.00 (93.75%)	20,408.00 (92.93%)	
Smoking status					
Never	37,226.00 (56.50%)	12,113.00 (55.16%)	12,555.00 (57.17%)	12,558.00 (57.18%)	< 0.001
Previous	23,148.00 (35.14%)	7573.00 (34.48%)	7755.00 (35.31%)	7820.00 (35.61%)	
Current	5508.00 (8.36%)	2275.00 (10.36%)	1650.00 (7.51%)	1583.00 (7.21%)	
Physical activity					
Non	24,126.00 (36.62%)	8734.00 (39.77%)	8206.00 (37.37%)	7186.00 (32.72%)	< 0.001
Yes	41,756.00 (63.38%)	13,227.00 (60.23%)	13,754.00 (62.63%)	14,775.00 (67.28%)	
Education					
Unknown	6847.00 (10.39%)	2886.00 (13.14%)	2006.00 (9.13%)	1955.00 (8.90%)	< 0.001
College	25,698.00 (39.01%)	7418.00 (33.78%)	8987.00 (40.92%)	9293.00 (42.32%)	
Other levels	33,337.00 (50.60%)	11,657.00 (53.08%)	10,967.00 (49.94%)	10,713.00 (48.78%)	
BMI					
< 30	51,080.00 (77.53%)	16,768.00 (76.35%)	17,233.00 (78.47%)	17,079.00 (77.77%)	< 0.001
≥ 30	14,802.00 (22.47%)	5193.00 (23.65%)	4727.00 (21.53%)	4882.00 (22.23%)	
TDI					
Median (Q1, Q3)	−1.95 (−3.47, 0.42)	−1.83 (−3.38, 0.67)	−2.04 (−3.55, 0.23)	−1.97 (−3.47, 0.37)	< 0.001
Diet score					
Mean (SD)	2.84 (1.29)	2.65 (1.23)	2.81 (1.27)	3.05 (1.33)	< 0.001
Sleep score					
Median (Q1, Q3)	1.00 (1.00, 2.00)	1.00 (1.00, 2.00)	1.00 (1.00, 2.00)	1.00 (1.00, 2.00)	0.064
CRP					
Median (Q1, Q3)	1.21 (0.61, 2.51)	1.31 (0.64, 2.75)	1.17 (0.60, 2.41)	1.16 (0.59, 2.37)	< 0.001
Neutrophil					
Mean (SD)	4.20 (1.37)	4.24 (1.40)	4.18 (1.35)	4.17 (1.36)	< 0.001
WBC					
Mean (SD)	6.84 (2.03)	6.92 (2.09)	6.81 (1.91)	6.81 (2.08)	< 0.001
FEV1					
Mean (SD)	2.89 (0.76)	2.86 (0.76)	2.90 (0.76)	2.90 (0.76)	< 0.001
FVC					
Mean (SD)	3.80 (0.97)	3.76 (0.96)	3.81 (0.97)	3.83 (0.98)	< 0.001
PM2.5					
Mean (SD)	9.85 (0.93)	9.84 (0.92)	9.88 (0.92)	9.84 (0.93)	< 0.001
NO_2_					
Mean (SD)	26.78 (7.50)	26.66 (7.50)	26.96 (7.45)	26.73 (7.54)	< 0.001
Hypertension					
No	46,550.00 (70.66%)	15,361.00 (69.95%)	15,718.00 (71.58%)	15,471.00 (70.45%)	< 0.001
Yes	19,332.00 (29.34%)	6600.00 (30.05%)	6242.00 (28.42%)	6490.00 (29.55%)	
Hyperlipidemia					
No	55,505.00 (84.25%)	18,374.00 (83.67%)	18,588.00 (84.64%)	18,543.00 (84.44%)	0.012
Yes	10,377.00 (15.75%)	3587.00 (16.33%)	3372.00 (15.36%)	3418.00 (15.56%)	
IHD					
No	59,064.00 (89.65%)	19,591.00 (89.21%)	19,735.00 (89.87%)	19,738.00 (89.88%)	0.031
Yes	6818.00 (10.35%)	2370.00 (10.79%)	2225.00 (10.13%)	2223.00 (10.12%)	
ACOS					
No	65,150.00 (98.89%)	21,672.00 (98.68%)	21,771.00 (99.14%)	21,707.00 (98.84%)	< 0.001
Yes	732.00 (1.11%)	289.00 (1.32%)	189.00 (0.86%)	254.00 (1.16%)	
Median follow‐up time					
Mean (SD)	12.45 (1.64)	12.42 (1.69)	12.46 (1.62)	12.46 (1.63)	0.007

Abbreviations: ACOS, asthma‐chronic obstructive pulmonary disease overlap syndrome; BMI, body mass index; CRP, C‐reactive protein; FEV1, forced expiratory volume in 1 s; FVC, forced vital capacity; IHD, ischemic heart disease; TDI, Townsend Deprivation Index; WBC, white blood cell.

^a^ *n* (%).

^b^ One‐way analysis of means (not assuming equal variances); Pearson's Chi‐squared test.

### 3.2 Association Between CDAI and ACOS

We constructed three models to explore the association of CDAI with ACOS (Table 2). Model 1 adjusted for age, gender, and ethnicity, while Model 2 further adjusted for drinking status, smoking status, and physical activity on the basis of Model 1. Model 3 adjusted for all covariates. In Model 3, no statistically significant association was found between CDAI and ACOS [1.01 (0.94, 1.09)]. Further sensitivity analysis using T2 as a reference revealed that the association between CDAI and ACOS showed a nonlinear trend of first decreasing and then increasing, with HR (95% CI) of 1.33 (1.11, 1.61) at T1 and 1.36 (1.13, 1.65) at T3.

**TABLE 2:** Association between composite dietary antioxidant index and ACOS.

	Model 1 HR (95% CI)	Model 2 HR (95% CI)	Model 3 HR (95% CI)
Asthma			
CDAI	0.93 (0.86, 1.00)	0.97 (0.90, 1.04)	1.01 (0.94, 1.09)
CDAI category			
Tertile 2	1.0	1.0	1.0
Tertile 1	1.62 (1.35, 1.95)	1.50 (1.25, 1.80)	1.33 (1.11, 1.61)
Tertile 3	1.34 (1.11, 1.62)	1.36 (1.13, 1.64)	1.36 (1.13, 1.65)
Gender			
Male	0.99 (0.88, 1.10)	1.01 (0.91, 1.13)	1.06 (0.95, 1.17)
Female	0.88 (0.79, 0.98)	0.93 (0.83, 1.03)	0.98 (0.88, 1.08)
Age			
< 60	0.92 (0.81, 1.05)	0.97 (0.85, 1.11)	1.03 (0.91, 1.16)
≥ 60	0.93 (0.85, 1.02)	0.96 (0.88, 1.05)	1.01 (0.92, 1.10)
BMI			
< 30	0.94 (0.85, 1.03)	0.98 (0.89, 1.08)	1.04 (0.95, 1.14)
≥ 30	0.92 (0.82, 1.04)	0.95 (0.84, 1.06)	0.98 (0.87, 1.10)

*Note:* Model 1: Age, gender, and ethnicity were adjusted. Model 2: Age, gender, ethnicity, drinking status, smoking status, and physical activity were adjusted. Model 3: Age, gender, ethnicity, drinking status, smoking status, physical activity, education, BMI, TDI, diet score, sleep score, CRP, neutrophil, WBC, FEV1, FVC, PM2.5, NO2, IHD, hypertension, and hyperlipidemia were adjusted. In the analysis stratified by gender, age, and BMI, the model is not adjusted for the stratification variable itself.

**FIGURE 2:**
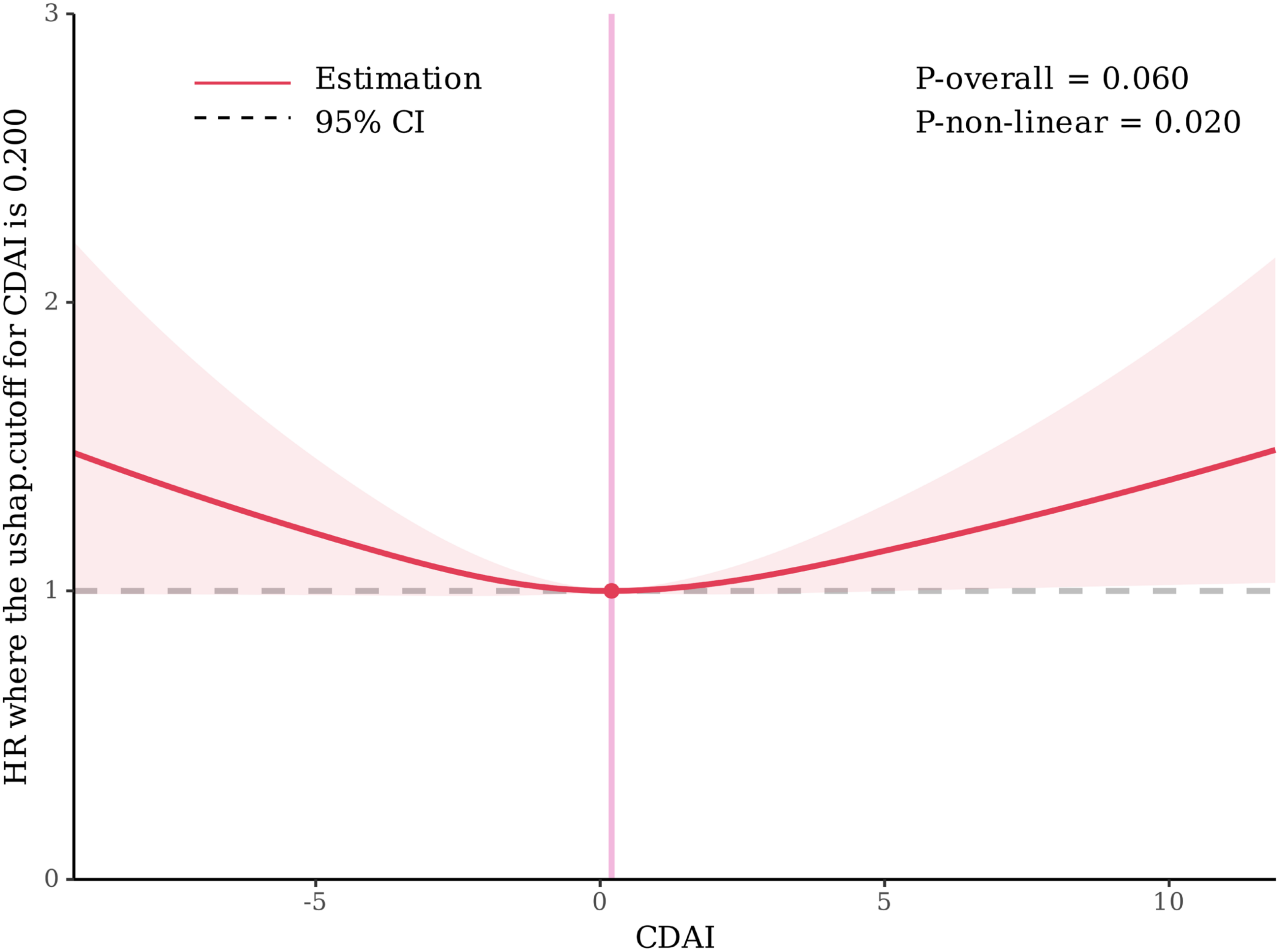
Association of composite dietary antioxidant index with ACOS. Age, gender, ethnicity, drinking status, smoking status, physical activity, education, BMI, TDI, diet score, sleep score, CRP, neutrophil, WBC, FEV1, FVC, PM_2.5_, NO_2_, IHD, hypertension, and hyperlipidemia were adjusted.

**FIGURE 3:**
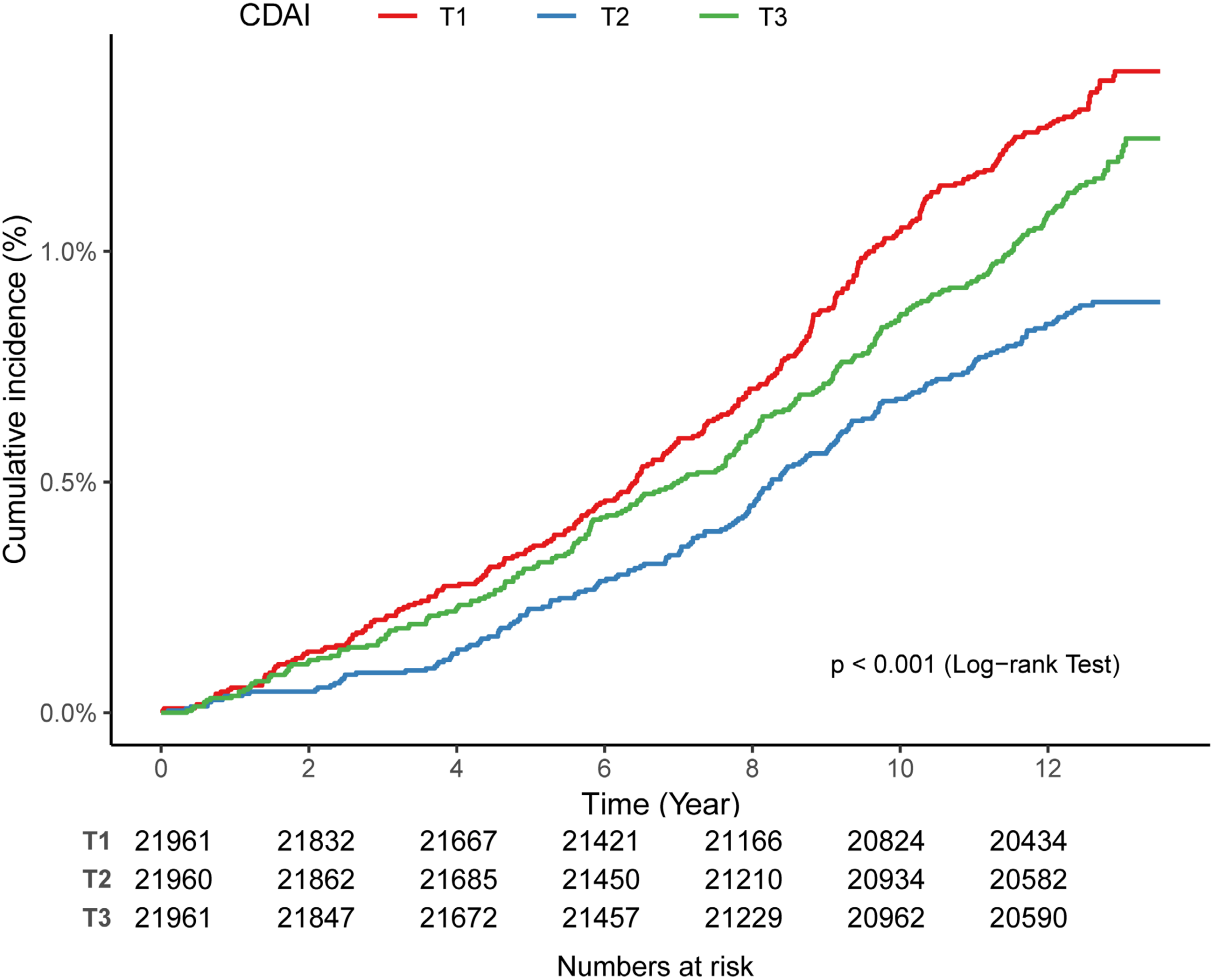
The Kaplan–Meier analysis for ACOS was based on composite dietary antioxidant index.

To determine whether a nonlinear association existed, we performed RCS. The result confirmed the nonlinear association of CDAI with ACOS (Figure 2). The recursive algorithm identified the inflection point of the association between CDAI and ACOS as 0.2 (Table 3). Before the inflection point, CDAI was negatively correlated with ACOS [0.81 (0.69, 0.95)], and after the inflection point, CDAI was positively associated with ACOS [1.14 (1.02, 1.27)]. The results of the competing risk model were generally consistent with those from the primary Cox proportional hazards model. People at the T2 level had the lowest risk of ACOS in the association between CDAI and ACOS (Figure 3).

**TABLE 3:** Threshold effect analysis of composite dietary antioxidant index with ACOS using a two‐piecewise linear regression model.

	Adjust HR (95% CI)	*p*
ACOS		
Fitting by standard linear model	1.01 (0.94, 1.09)	0.731
Fitting by two‐piecewise linear model		
Inflection point	0.2	
0.2	0.81 (0.69, 0.95)	0.012
0.2	1.14 (1.02, 1.27)	0.019
Log‐likelihood ratio	< 0.001	

*Note:* Age, gender, ethnicity, drinking status, smoking status, physical activity, education, BMI, TDI, diet score, sleep score, CRP, neutrophil, WBC, FEV1, FVC, PM2.5, NO2, IHD, hypertension, and hyperlipidemia were adjusted.

### 3.3 Subgroup Analysis

**FIGURE 4:**
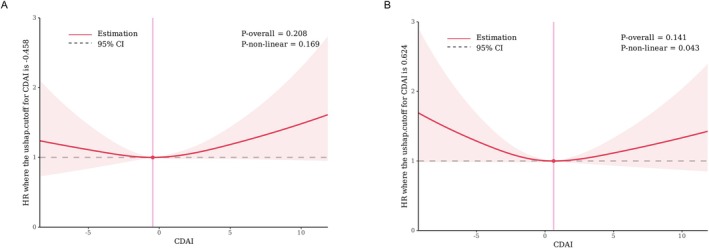
Association between composite dietary antioxidant index and ACOS stratified by sex (A) Male; (B) Female.

**FIGURE 5:**
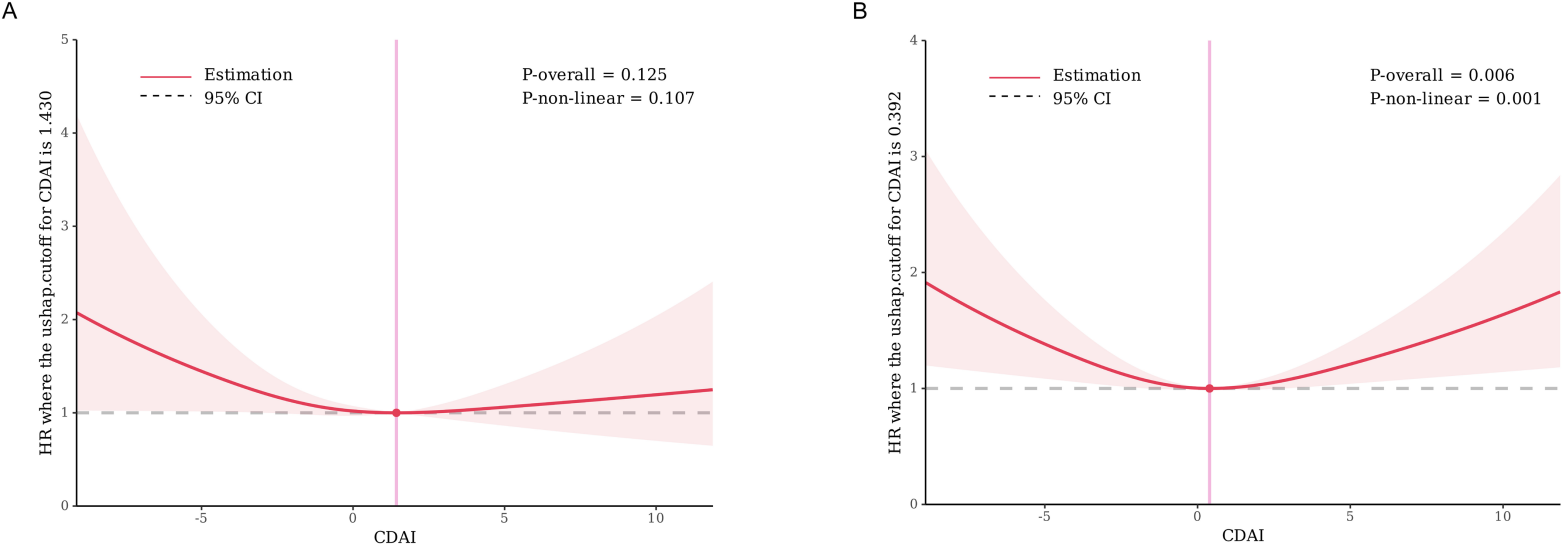
Association between composite dietary antioxidant index and ACOS stratified by age (A: < 60; B: ≥ 60).

**FIGURE 6:**
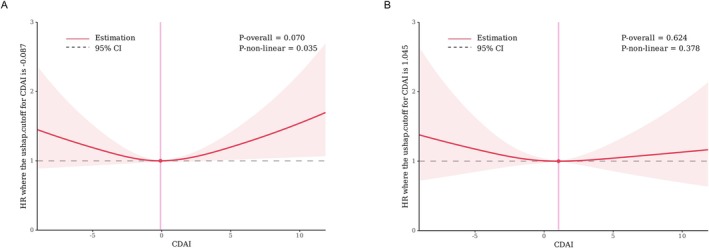
Association between composite dietary antioxidant index and ACOS stratified by BMI (A) < 30; (B) ≥ 30.

**FIGURE 7:**
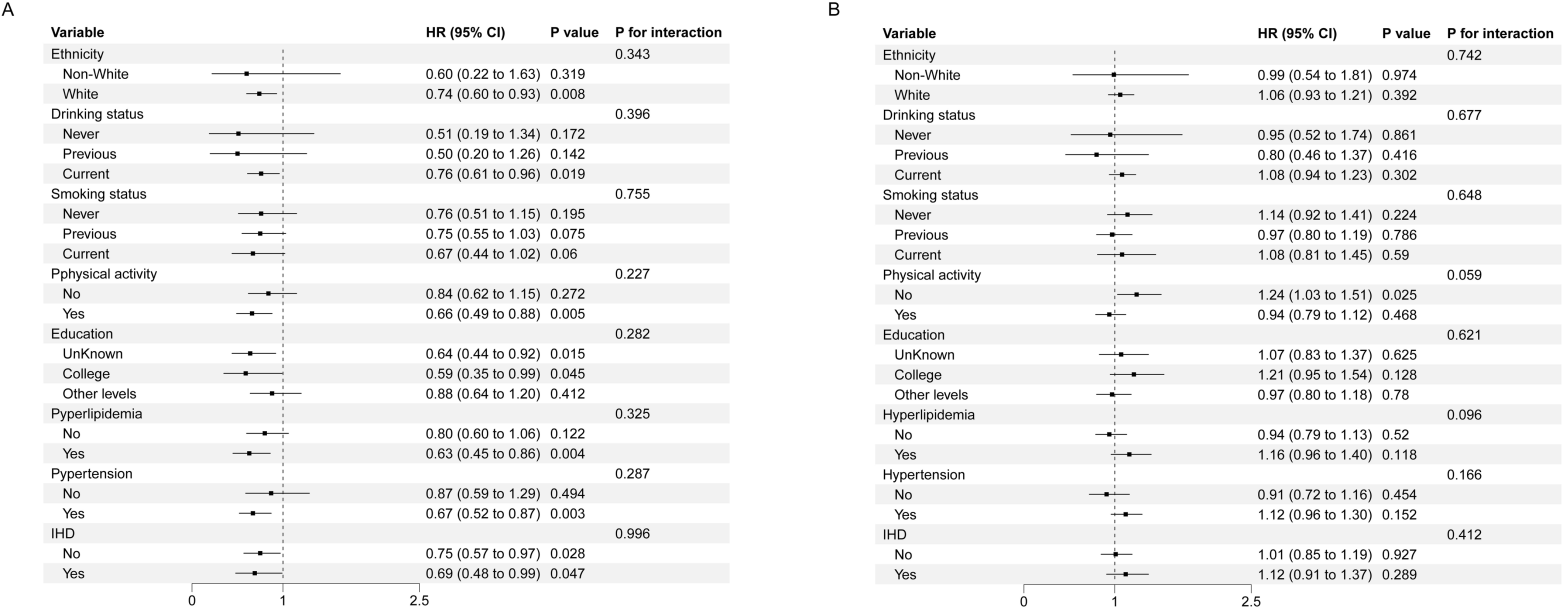
Subgroup analysis of association between composite dietary antioxidant index with ACOS (A: CDAI < 0.2; B: CDAI > 0.2).

We conducted the subgroup analysis to explore whether there were differences between subgroups. In the stratified analysis by gender, we found that after adjusting for all covariates, the association between CDAI and ACOS was not statistically significant, with HR (95% CI) of 1.06 (0.95, 1.17) in males and 0.98 (0.88, 1.08) in females (Table 2). Similarly, no significant associations were found in age and BMI subgroups (Table 2). Subsequent RCS analysis revealed a nonlinear association only in females, participants aged ≥ 60 and with BMI < 30 (Figures 4, 5, 6). Further subgroup analysis for other categorical variables revealed no significant interactions (Figure 7).

### 3.4 Sensitivity Analysis

To assess the robustness of the results, we conducted further sensitivity analyses. After excluding participants who developed ACOS during the 1‐ and 2‐year follow‐up periods, no significant association was observed between CDAI and the risk of ACOS; however, when categorized by tertiles, the risk of ACOS remained significantly elevated in the low‐ and high‐CDAI groups compared with the group with moderate CDAI levels, and the direction of the association was consistent with that of the main analysis, suggesting that the results are unlikely to be driven entirely by early‐follow‐up events or potential reverse causality (Table S1). Furthermore, after excluding CDAI outliers, the aforementioned associations by tertile remained relatively stable, indicating that the study results were not dominated by a small number of outliers (Table S2). The RCS analysis further demonstrated that, after excluding early events and outliers, the nonlinear association between CDAI and the risk of ACOS persisted (Figures S2 and S4). Kaplan–Meier curves also showed significant differences in the cumulative incidence of ACOS across different CDAI tertiles, with the group with moderate CDAI exhibiting a relatively lower cumulative incidence (Figures S3 and S5). Overall, the results of the sensitivity analyses were largely consistent with the main analysis, supporting the existence of a robust nonlinear association between CDAI and the risk of ACOS.

## 4 Discussion

This study was the first cohort study to explore the association of CDAI with ACOS. After adjusting for all covariates, the association between CDAI and ACOS was found to be nonlinear, and the association before and after the inflection point was further calculated. The association of CDAI with ACOS showed an opposite association before and after the inflection point. Further stratification analysis revealed that the nonlinear association existed only in females, participants aged ≥ 60, and with BMI < 30.

This study revealed that both excessively high or low CDAI were significantly associated with an increased risk of ACOS, which provided new insights into the role of antioxidant‐rich dietary patterns in respiratory health. This finding suggests that moderately increasing antioxidant intake may bring significant lung function protection benefits in high‐risk populations with low dietary antioxidant levels. However, once a certain threshold is exceeded, higher intake does not necessarily provide additional benefits, which is conducive to individualized nutritional guidance and avoids the potentially adverse effects of high‐dose supplementation. At the same time, in the long‐term management of ACOS, CDAI levels can be monitored through dietary assessment, and timely intervention can be carried out before the inflection point to delay disease progression. At the public health care level, this finding may serve as a useful reference for future research on dietary recommendations. Rather than generally encouraging a high‐antioxidant diet, interventions should focus on people with insufficient intake to improve resource utilization efficiency. Finally, this study emphasizes the concept of “moderate antioxidants,” which can provide a reference for health education and dietary guideline revisions to reduce the public's blind pursuit of high‐dose antioxidant supplements.

Current research on the association of CDAI with respiratory diseases has largely focused on either asthma or COPD alone, and the results were mostly inconsistent. One study in adults showed that CDAI was negatively associated with asthma (Chen et al. 2025), but another study in children aged 3–18 years did not reveal any association between CDAI and asthma (Tian et al. 2024). Two cross‐sectional studies from the United States found a L‐shaped association between CDAI and COPD (Xu et al. 2024; Wu et al. 2024). No subgroup differences were found in their studies. In contrast to the above studies, another study showed that the negative association between CDAI and COPD was linear (Xiang and Wang 2025), and another research on adults over 40 years old confirmed this result and further explored the mediating role of inflammatory markers (Liu, Li, et al. 2023).

The mechanism of the nonlinear association of CDAI with ACOS may be related to the “threshold effect” of antioxidant defense. Low CDAI represents insufficient dietary antioxidant intake and reduced activity of the body's endogenous antioxidant system (such as glutathione, superoxide dismutase, catalase, etc.), which is unable to effectively clear the excess ROS produced by the inflammatory response. The excessive accumulation of ROS can damage lung tissue by oxidizing DNA, proteins, and lipid membranes, activate inflammation‐related signaling pathways, and promote the sustained release of inflammatory mediators, thereby accelerating the onset and progression of asthma and COPD (Kirkham and Rahman 2006; Sahiner et al. 2018). At this stage, any increase in dietary antioxidants can significantly reduce the level of oxidative stress, thus showing a negative association. However, when the association of CDAI is greater than the threshold, the body's antioxidant system is close to saturation, ROS is maintained at a steady‐state level, and the marginal effect of additional antioxidant intake is reduced. Furthermore, excessively high antioxidant loads may paradoxically lead to “antioxidant‐oxidative stress imbalance,” inducing reductive stress. This state disrupts intracellular redox homeostasis, interferes with mitochondrial function, causes abnormal ROS metabolism, and damages alveolar epithelial cells and airway epithelial barriers, thereby accelerating COPD‐related tissue structural damage (Rahman and Adcock 2006).

This study revealed that the association of CDAI with asthma and COPD was nonlinear only in females. The underlying mechanisms for this gender difference remain unclear. Previous studies suggest that gender differences in sex hormone levels, immune responses, and nutritional metabolism may, to some extent, influence the relationship between antioxidant‐related exposure and respiratory outcomes. Specifically, estrogen in female subjects has certain antioxidant and anti‐inflammatory effects (Mann et al. 2007), but its levels fluctuate with the menstrual cycle or menopause (Strehlow et al. 2003), making females more susceptible to the amplifying effects of oxidative stress and inflammatory responses when their diets are deficient in antioxidants. Additionally, women's immune systems are generally more active and respond more strongly to inflammatory mediators (Camon et al. 2024). Combined with their unique utilization of antioxidant nutrients such as vitamin C, vitamin E, and carotenoids, this makes them more sensitive to changes in antioxidant levels (Ortona et al. 2019). In contrast, men lack similar hormonal protection, have relatively mild immune responses, and are less dependent on dietary antioxidants due to higher iron reserves and differences in airway structure, making it difficult for them to benefit from the CDAI. The mechanisms above represent possible biological explanations. Further research combining more precise biological measurements and basic research designs will be needed to elucidate the underlying mechanisms of these gender‐specific differences.

The present study holds the following distinct advantages over previous research. First, it was based on high‐quality research data from the UK Biobank, which had undergone rigorous standardization and quality control to ensure high reliability and minimize the impact of bias. Second, it featured the largest sample size to date, enhancing the statistical power and representativeness of the results. Third, it was a cohort study spanning over a decade, offering a higher level of evidence compared to previous cross‐sectional studies. Fourth, the outcomes of this study were derived from ICD codes for hospital diagnoses, while most previous studies used patient self‐reports for their outcomes, making this study more rigorous and credible. However, some limitations should also be noted. First, dietary data were self‐reported by participants and assessed only at baseline, which may introduce subjective bias and does not capture long‐term intake patterns. Further studies are needed to investigate how longitudinal changes in CDAI over time may influence the risk of ACOS. Second, the participants in the UK Biobank were from the UK and may not fully represent other regions or ethnic groups. The generalizability and applicability of the study results require further confirmation. Third, given the observational design of this study, it is not possible to draw conclusions regarding causality. Fourth, no globally standardized diagnostic criteria have been established for ACOS. This diagnostic heterogeneity may limit comparability across studies and result in differential performance of the same biomarker across study populations. Further research is needed to investigate the relationship between exposure and outcomes under different diagnostic criteria. Finally, although this study has relatively comprehensively included confounding factors that require adjustment, the influence of certain uncollected potential confounding factors on the results cannot be avoided.

## 5 Conclusion

In this large‐scale prospective cohort study, we found a nonlinear association between CDAI and ACOS and further revealed subgroup differences. This study may provide a reference for alleviating respiratory diseases through comprehensive antioxidant dietary intake. Further randomized controlled trials are needed to verify the stability of the study results.

## Author Contributions

**Ming Lei:** funding acquisition, supervision, project administration, writing – review and editing. **Xixue Lu:** data curation, formal analysis. **Chunli Zeng:** methodology, writing – original draft. **Lihong Lou:** visualization, validation, methodology. **Hequn Lyu:** methodology, writing – original draft.

## Funding

This work was supported by National Construction Program for TCM Advantageous Specialties (Department of Critical Care Medicine), Pudong New Area “National Pilot Zone for Inheritance and Innovative Development of Traditional Chinese Medicine” Construction Plan (PDZY‐2‐25‐0713), Jiangsu Provincial Traditional Chinese Medicine Science and Technology Development Plan Project (No. MS2023130), National Project for Clinical Collaboration between Chinese Medicine and Western Medicine on Major and Difficult Diseases (Electro‐Chemo‐Thermal Injury), and Nanjing University of Chinese Medicine Natural Science Foundation General Project (XZR2023096).

## Ethics Statement

The approval for the UKB project was obtained from the North West Research Ethics Committee (11/NW/0382) and informed consents were signed by all participants.

## Conflicts of Interest

The authors declare no conflicts of interest.

## Supporting information


**Table S1:** Association between composite dietary antioxidant index and ACOS.
**Table S2:** Association between composite dietary antioxidant index and ACOSS (Excluding outliers).
**Figure S1:** Proportional hazards assumptions.
**Figure S2:** Association between composite dietary antioxidant index and ACOS (A: Excluding events within 1 year, B: Excluding events within 2 years).
**Figure S3:** The Kaplan–Meier analysis for ACOS was based on composite dietary antioxidant index (A: Excluding events within 1 year, B: Excluding events within 2 years).
**Figure S4:** Association between composite dietary antioxidant index and ACOS (Excluding outliers).
**Figure S5:** Association between composite dietary antioxidant index and ACOS (Excluding outliers).

## Data Availability

The data that support the findings of this study are available from the UK Biobank. Restrictions apply to the availability of these data, which were used under license for this study. Data are available from https://www.ukbiobank.ac.uk with the permission of the UK Biobank. This research has been conducted using the UK Biobank Resource under Application Number 552996.
