# Supplementary material for: Association Between Composite Dietary Antioxidant Index and Asthma‐Chronic Obstructive Pulmonary Disease Overlap Syndrome: Evidence From a Large Cohort in the UK Biobank
Source: Food Sci Nutr. 2026 Jul 26;14(7):e72153. doi: 10.1002/fsn3.72153 (PMC13402016; doi:10.1002/fsn3.72153)
Supplement: Supplementary file 1 — Table S1: Association between composite dietary antioxidant index and ACOS. Table S2: Association between composite dietary antioxidant index and ACOSS (Excluding outliers). Figure S1: Proportional hazards assumptions. Figure S2: Association between composite dietary antioxidant index and ACOS (A: Excluding events within 1 year, B: Excluding events within 2 years). Figure S3: The Kaplan–Meier analysis for ACOS was based on composite dietary antioxidant index (A: Excluding events within 1 year, B: Excluding events within 2 years). Figure S4: Association between composite dietary antioxidant index and ACOS (Excluding outliers). Figure S5: Association between composite dietary antioxidant index and ACOS (Excluding outliers). [file FSN3-14-e72153-s001.docx]

**Supplementary Table 1.** Association between composite dietary antioxidant index and ACOS

| Analysis | Exposure | Model 1 HR (95% CI) | Model 2 HR (95% CI) | Model 3 HR (95% CI) |
| --- | --- | --- | --- | --- |
| Excluding events within 1 year | CDAI, continuous | 0.98 (0.96, 1.00) | 1.00 (0.98, 1.02) | 1.01 (0.98, 1.03) |
| Excluding events within 1 year | Tertile 1 | 1.61 (1.33, 1.94) | 1.40 (1.16, 1.69) | 1.33 (1.10, 1.61) |
| Excluding events within 1 year | Tertile 2 | 1.00 (Reference) | 1.00 (Reference) | 1.00 (Reference) |
| Excluding events within 1 year | Tertile 3 | 1.34 (1.11, 1.63) | 1.38 (1.14, 1.67) | 1.37 (1.13, 1.66) |
| Excluding events within 2 years | CDAI, continuous | 0.98 (0.96, 1.01) | 1.00 (0.98, 1.02) | 1.01 (0.99, 1.03) |
| Excluding events within 2 years | Tertile 1 | 1.53 (1.26, 1.85) | 1.33 (1.10, 1.62) | 1.27 (1.04, 1.54) |
| Excluding events within 2 years | Tertile 2 | 1.00 (Reference) | 1.00 (Reference) | 1.00 (Reference) |
| Excluding events within 2 years | Tertile 3 | 1.27 (1.04, 1.54) | 1.30 (1.07, 1.58) | 1.29 (1.06, 1.58) |

Model 1: Age, gender, and ethnicity were adjusted.

Model 2: Age, gender, ethnicity, drinking status, smoking status, and physical activity were adjusted.

Model 3: Age, gender, ethnicity, drinking status, smoking status, physical activity, education, BMI, TDI, diet score, sleep score, CRP, neutrophil, WBC, FEV1, FVC, PM2.5, NO2, IHD, hypertension, and hyperlipidemia were adjusted.

**Supplementary Table 2.** Association between composite dietary antioxidant index and ACOSS(Excluding outliers).

| Exposure | Model 1 HR (95% CI) | Model 2 HR (95% CI) | Model 3 HR (95% CI) |
| --- | --- | --- | --- |
| CDAI, continuous | 0.98 (0.96, 1.00); | 1.00 (0.98, 1.02); | 1.00 (0.98, 1.02); |
| Tertile 1 | 1.61 (1.34, 1.94); | 1.40 (1.16, 1.68); | 1.33 (1.10, 1.60); |
| Tertile 2 | 1.00 (Reference) | 1.00 (Reference) | 1.00 (Reference) |
| Tertile 3 | 1.32 (1.09, 1.60); | 1.36 (1.12, 1.64); | 1.34 (1.11, 1.63); |

Model 1: Age, gender, and ethnicity were adjusted.

Model 2: Age, gender, ethnicity, drinking status, smoking status, and physical activity were adjusted.

Model 3: Age, gender, ethnicity, drinking status, smoking status, physical activity, education, BMI, TDI, diet score, sleep score, CRP, neutrophil, WBC, FEV1, FVC, PM2.5, NO2, IHD, hypertension, and hyperlipidemia were adjusted.


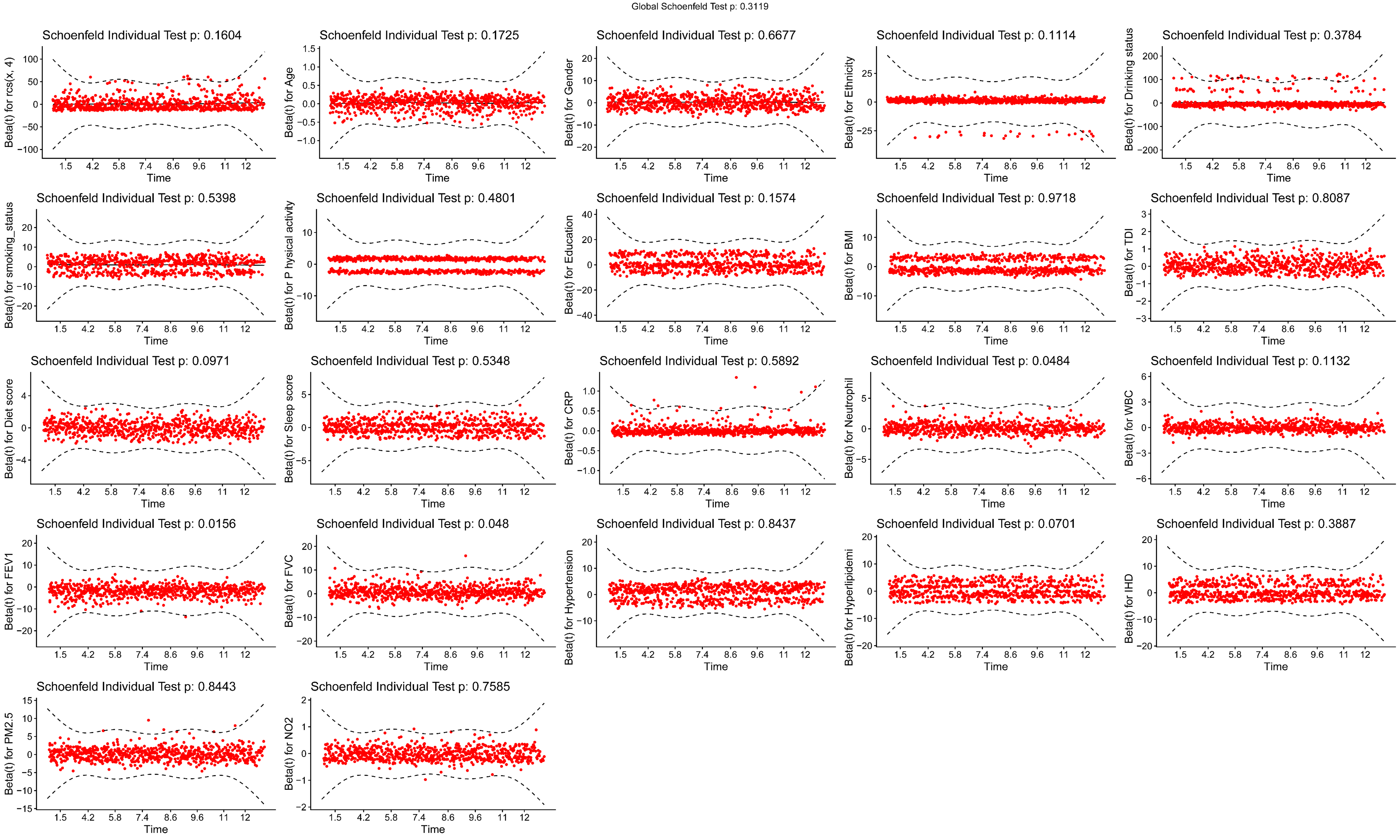


**Supplementary Figure 1.** Proportional hazards assumptions.


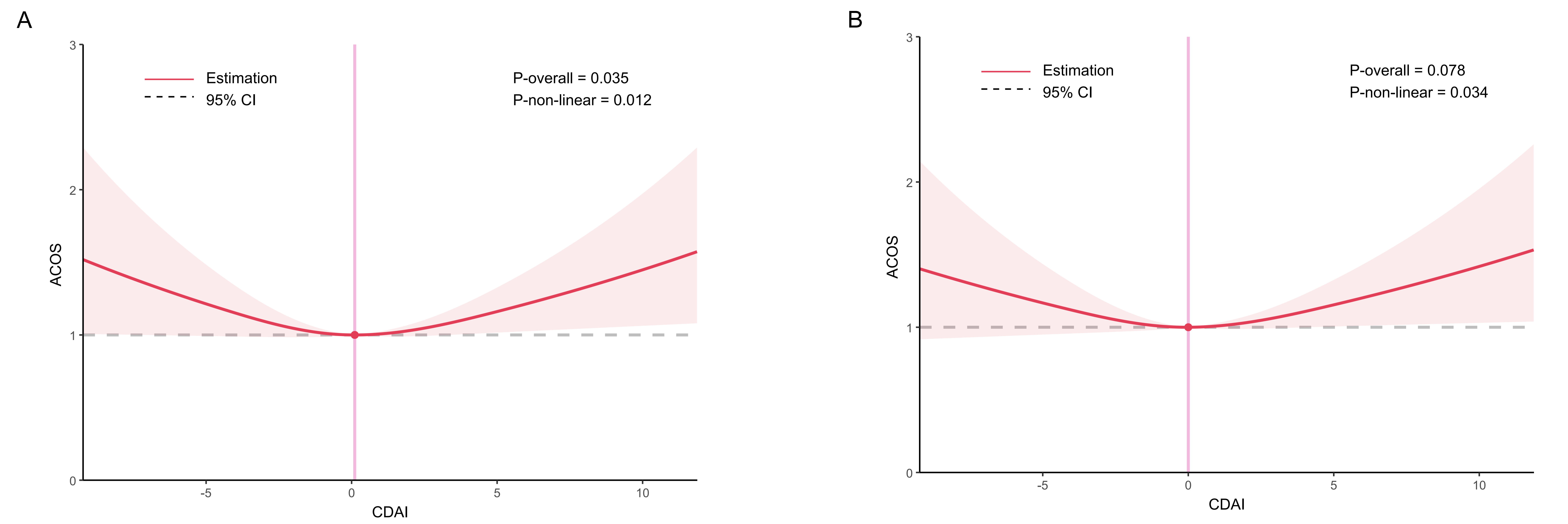


**Supplementary Figure 2.** Association between composite dietary antioxidant index and ACOS(A:Excluding events within 1 year,B:Excluding events within 2 years).


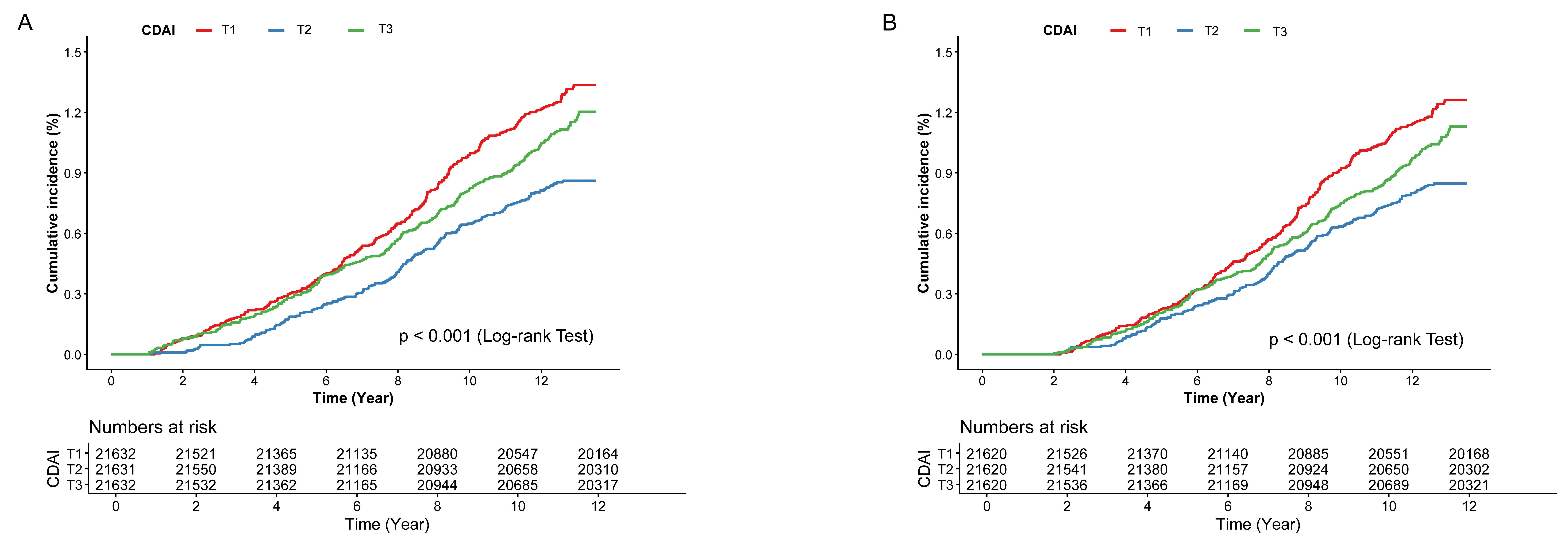


**Supplementary Figure 3.** The Kaplan–Meier analysis for ACOS was based on composite dietary antioxidant index(A:Excluding events within 1 year,B:Excluding events within 2 years).


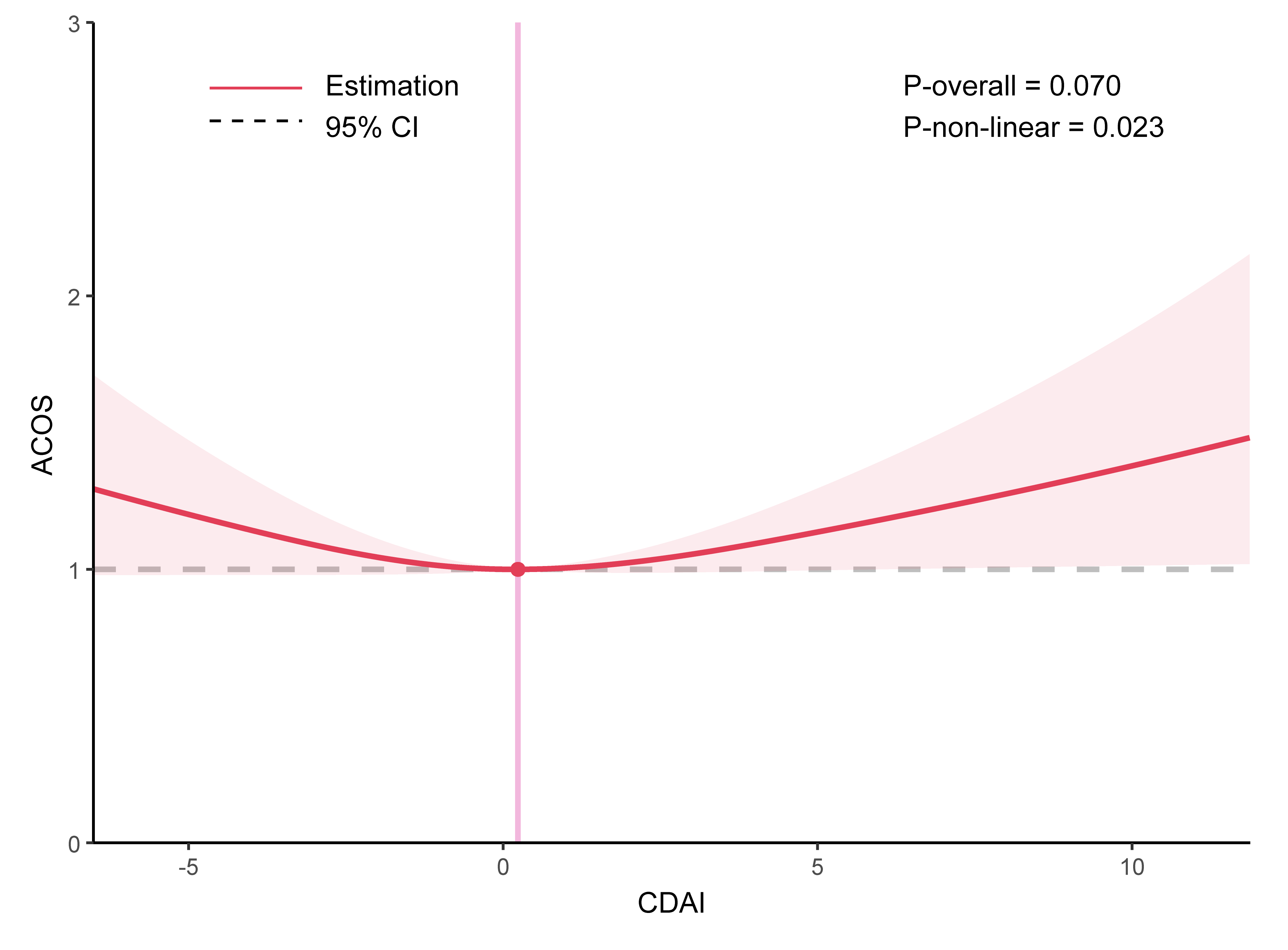


**Supplementary Figure 4** Association between composite dietary antioxidant index and ACOS(Excluding outliers).


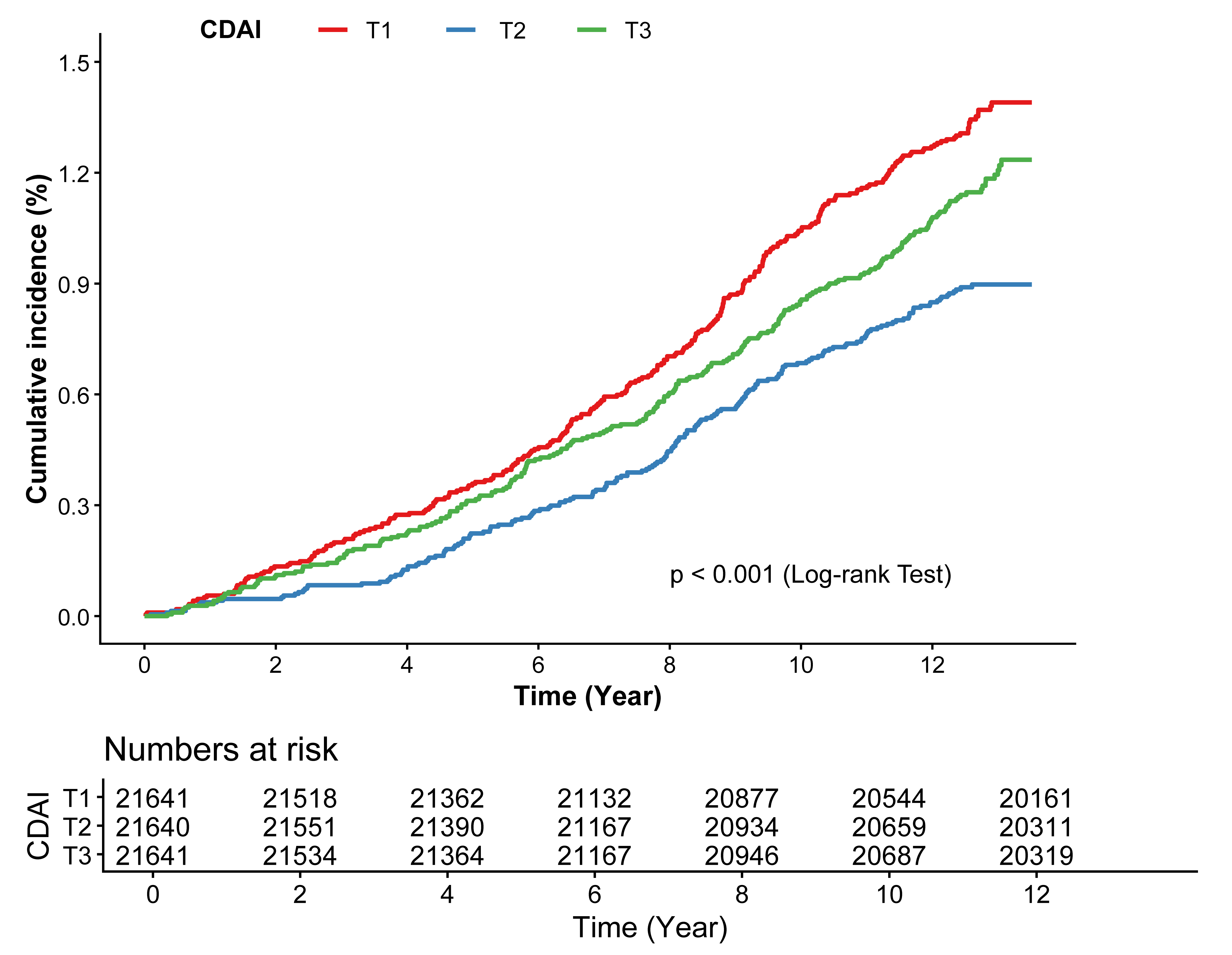


**Supplementary Figure 5.** Association between composite dietary antioxidant index and ACOS(Excluding outliers).
